# Prevalence and risk of new-onset diabetes mellitus after COVID-19: a systematic review and meta-analysis

**DOI:** 10.3389/fendo.2023.1215879

**Published:** 2023-09-04

**Authors:** Chiara Bellia, Aikaterini Andreadi, Ilenia D’Ippolito, Letizia Scola, Sonia Barraco, Marco Meloni, Davide Lauro, Alfonso Bellia

**Affiliations:** ^1^ Department of Biomedicine, Neurosciences, and Advanced Diagnostics, University of Palermo, Palermo, Italy; ^2^ Transfusion Medicine Unit, University Hospital “Paolo Giaccone”, Palermo, Italy; ^3^ Department of Systems Medicine, University of Rome Tor Vergata, Rome, Italy; ^4^ Division of Endocrinology and Diabetes, Fondazione Policlinico Tor Vergata, Rome, Italy

**Keywords:** diabetes, COVID – 19, incidence, systematic review and meta-analysis, new onset diabetes, SARS-CoV2 (COVID- 19)

## Abstract

**Aims:**

After the acute phase of SARS-CoV-2 infection, the onset of glycemic impairment and diabetes have been reported. Nevertheless, the exact burden of glycemic impairment and diabetes after COVID-19 has not been clearly described.

**Materials and methods:**

Electronic search was run in Pubmed (MEDLINE), Web of Science, Scopus, and ClinicalTrial.org for reports published from database inception to September 2022. We included observational studies reporting quantitative data on diabetes prevalence or its onset in subjects with a history of SARS-CoV-2 infection from at least 60 days. Risk of bias was assessed by the JBI’s critical appraisal checklist. Random effect model was used to calculate pooled data. The review protocol was registered on PROSPERO (CRD42022310722).

**Results:**

Among 1,630 records screened, 20 studies were included in the analysis. The mean or median age of participants ranged from ~ 35 to 64 years, with a percentage of males ranging from 28% to 80%. Only two studies were considered at low risk of bias. The estimate of diabetes prevalence, calculated on a total of 320,948 participants pooled with 38,731 cases, was 16% (95%CI: 11-22%). The estimate of proportion of incident cases of diabetes was 1.6% (95%CI: 0.8-2.7%). Subgroup analysis showed that previous hospitalization increased the prevalence of diabetes and the proportion of incident cases.

**Conclusion:**

Diabetes is common in individuals who have experienced SARS-CoV-2 infection, especially if they required hospitalization. This data may be helpful to screen for diabetes and manage its complications in individuals who experienced COVID-19.

**Systematic review registration:**

https://www.crd.york.ac.uk/prospero/display_record.php?ID=CRD42022310722, identifier CRD42022310722.

## Introduction

The COVID-19 pandemic is still today a global emergency and, after almost three years, its spread is far to be overcome. There are several clinical manifestations after SARS-CoV-2 infection, including medium and long-term COVID-19 sequelae, that need particular attention mostly because of their relationship with other comorbidities (1). It has been observed that advanced age and presence of comorbidities such as diabetes, hypertension and obesity are associated with the most severe forms of COVID-19 (2). Since the first reports on COVID-19 epidemic diabetic patients turned out to be at increased risk of acute complications after SARS-CoV-2 infection, and even at high risk to subsequently develop various and diverse symptoms characterizing the so-called “Long Covid syndrome” (3). Growing evidence also suggests that the numerous clinical abnormalities of long Covid might even extend to new onset diabetes (4).

The relationship between COVID-19 and diabetes can be therefore considered as bidirectional. On one hand, diabetic patients are at increased risk to be infected with SARS-CoV-2 and those with inadequate glycaemic control and chronic vascular complications have particularly high risk to develop the most severe form of the disease (5). On the other hand, there is also an increased risk of acute complications related to diabetes, considering both the pre-existing and new-onset manifestations (6), in patients with COVID-19 infection. Among the different mechanisms underlying this relationship, angiotensin-converting enzyme 2 (ACE-2) receptors could have a possible role due to their widespread localisation in key metabolic organs and tissues including pancreatic beta cells (7, 8). It has been demonstrated that SARS-CoV-2 virus enter in human cells after binding with ACE-2 receptors (9).

Growing evidence suggest that people infected with SARS-CoV-2 have increased risk of incident diabetes and incident use of antihyperglycemic therapy in the post-acute phase of the disease (4, 10), with a trend toward increasing risk according to pre-existing conventional risk factors for diabetes itself. This evidence is however not universally consistent, especially when looking at the time of onset of diabetes after SARS-CoV-2 infection (11).

According to this background, aims of our systematic review and meta-analyses were: (1) to assess the prevalence of diabetes and any types of gluco-metabolic abnormalities reported in patients been infected with SARS-CoV-2 after at least 60 days from the diagnosis; (2) to estimate the proportion of new onset diabetes after at least 60 days from the diagnosis of SARS-CoV-2 infection, in order to exclude all hyperglycaemias secondary to steroids use or related to the acute phase of the disease.

## Methods

### Reporting and study protocol registration

This review was conducted and reported according to the PRISMA statement (**Supplementary S1**) (12). The study protocol was registered in the PROSPERO register before starting literature search (CRD42022310722).

### Inclusion and exclusion criteria

The review question was: which is the prevalence of metabolic impairment (any grade) and diabetes in patients with a history of SARS-CoV-2 infection? Due to the nature of the systematic review (epidemiological), we considered Condition, Context, and Population to organize our review question instead of the well-established PICO framework, specifically intended for intervention studies (13). We included studies evaluating the following conditions: a) diabetes, defined according to American Diabetes Association 2021 diagnostic criteria, or the need for oral antidiabetic treatment and/or insulin; b) dysglycemia, defined as impaired fasting glucose (fasting plasma glucose 100-126 mg/dl), and/or impaired glucose tolerance (2h-plasma glucose 140-199 mg/dl), and/or HbA1c 5.7-6.4%; c) HbA1c > 7% or any measure of high glycemic variability in patients with a diagnosis of diabetes before SARS-CoV-2 infection. In an initial version of the experimental design, we had also considered as inclusion criteria the need for oral antidiabetic treatment intensification, the new need or increase in the insulin dose and the appearance of diabetes-related complications, in order to identify the use of insulin therapy or the development of complications as indicators of worsening diabetes after SARS-CoV-2 infection. However, we did not find any studies reporting such data. Therefore, since main objective of our meta-analysis was to find data on the prevalence and proportion of new cases of diabetes following the infection, we decided to remove these inclusion criteria in the final version of the manuscript. The population of interest was composed by patients with a history of SARS-CoV-2 infection, defined as a PCR positive test, irrespective of disease severity. No specific restrictions to the context were applied. In order to control the diabetogenic effect of corticosteroid therapy used for the treatment of acute COVID-19, we excluded studies that assessed the condition within 60 days from SARS-CoV-2 infection.

Observational studies, including case series, prospective and retrospective cohort studies, cross-sectional studies, and randomized controlled trials were considered for inclusion. Reviews, editorials, comments, and any type of paper not reporting population estimates on diabetes and/or metabolic impairment were excluded from the quantitative synthesis. Only papers in English were considered.

### Search strategy and information sources

We searched Pubmed (MEDLINE), Web of Science, Scopus, and ClinicalTrial.org for reports published from database inception to the date of search. The databases were searched on 4 February 2022. Literature search was performed again on the 2 September 2022 to retrieve the most recent reports. The approach used to develop search strategy was adopted from intervention studies considering the non-experimental setting, and specifically defining the population of interest, the context, and the condition. The full search strategy used for MEDLINE was strictly adapted to search the other databases (**Supplementary S2**).

### Selection of studies

Duplicates were identified and removed by automation tools. Initially, four reviewers (CB, ID, SB, AB) independently reviewed the first 20 records and discussed inconsistency until consensus was reached. Then, two co-authors proceeded screening the remaining records by basing on title and abstract and working independently (CB, ID, SB, AB). No automation tools were used for study exclusion. Disagreement was resolved by consensus of reviewers. Full texts of the records that passed the first selection were screened by two co-authors working independently (CB, ID, SB, AB) to assure that inclusion criteria were fulfilled. In each step of records screening, the reviewer was blinded to the decision of the other one.

### Data collection process

Two reviewers extracted data independently into a standardized Excel format including any relevant

information about study design, setting, number of patients, demographics, symptoms and severity of COVID-19, time to diagnosis of COVID-19, prior history of diabetes, medications, history of glucocorticoids use, measures of dysglycemia, comorbidities. The form was piloted and calibration exercises on five records were conducted prior to formal data extraction to ensure consistency between reviewers. Again, disagreement between collectors was resolved by consensus. The overall diabetes cases as well as the new-onset ones were the conditions of interest.

### Study risk of bias assessment

Two reviewers (CB, ID) independently assessed the risk of bias for included studies using the Joanna Briggs Institute (JBI) checklist for prevalence studies (13). Briefly, this tool evaluates the quality of patient selection and condition ascertainment by a checklist, and give a score (maximum 9) to each study. We adopted the approach provided by Naing et al. (14) to evaluate sample size. Critical appraisal of the selected study is reported in **Supplementary S2**.

### Statistical analysis and synthesis methods

Pooled estimates of prevalence and 95% confidence intervals were calculated by a random effect model using the Der Simonian and Laird method; and double arcsine transformations were applied to stabilize the variance. Both prevalent and incident cases were included. The overall and subgroup pooled estimates were presented by a forest plot. Heterogeneity between the included studies was quantified by *I^2^* statistics. Subgroup analysis was carried out to detect potential sources of heterogeneity; studies were grouped together basing on hospitalization for COVID-19, duration of follow-up, and age of participants. Sensitivity analysis was performed to assess the robustness of results and it was conducted removing studies at high risk of bias, namely the studies with a score ≤ 5 at the critical appraisal. The presence of publication bias was addressed by funnel plot asymmetry and Begg’s test. All analysis were performed with MetaXL (15).

## Results

### Studies characteristics

**Figure 1:**
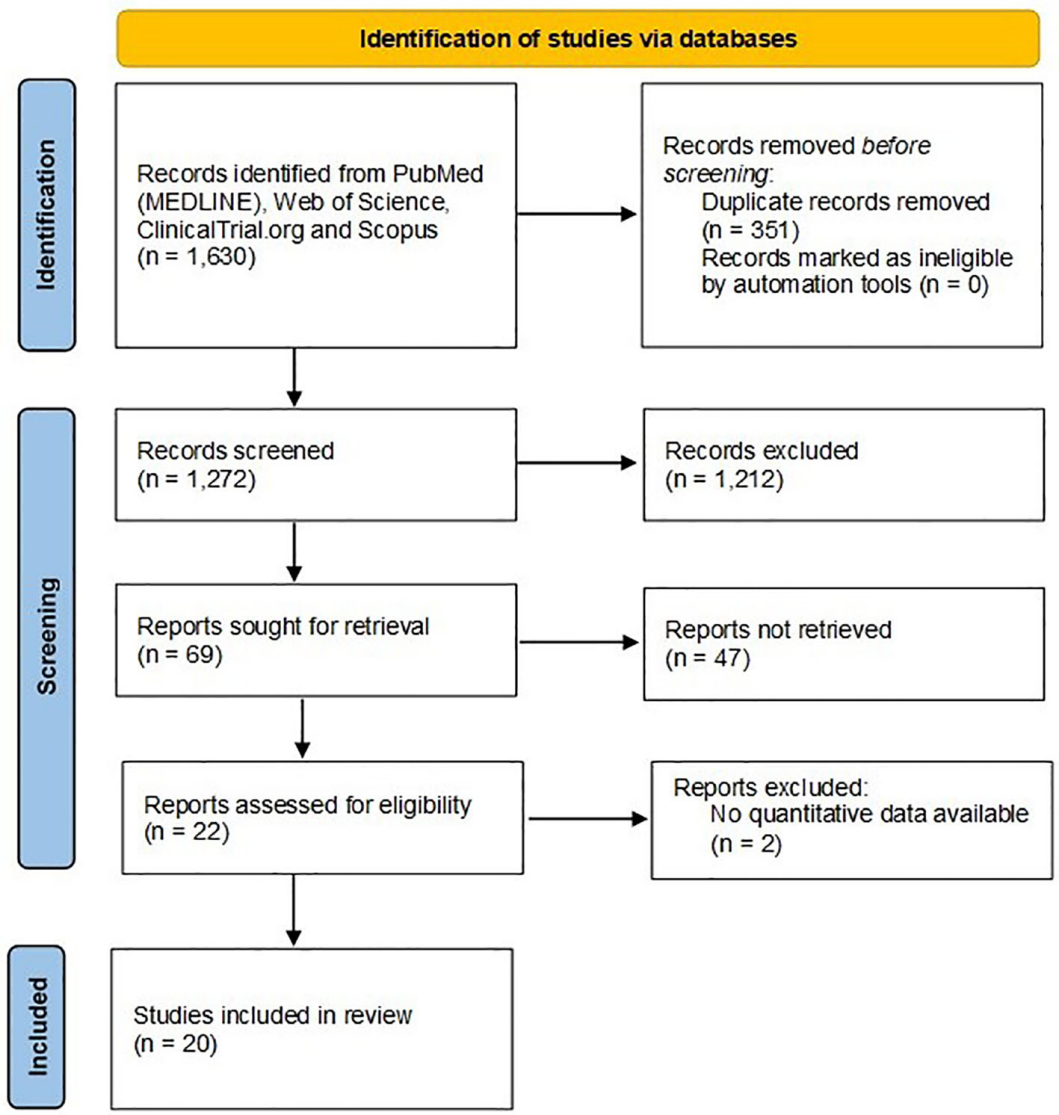
PRISMA 2020 flow chart for the record screening, review, and inclusion.

After removing duplicates, 1,272 abstracts were screened for inclusion. Sixty-nine full-text articles were assessed for eligibility. We excluded 47 of these because they did not fit inclusion criteria after revision of the full texts, thus leaving 22 publications to be included. Two articles were further excluded because no quantitative data were available after contacting the Authors (16, 17). Finally, 20 original paper were included in the review (4, 18–36), with the main characteristics of the studies displayed in **Table 1**. The detailed selection process is depicted in **Figure 1**. Overall, 3 studies had a cross-sectional design (15%), while 17 studies were longitudinal cohort studies (85%). Among cohort studies, 7 (42%) had a prospective design. The number of matched-cohort studies was 3 (17%). Overall, seven registry-based studies were included. Studies were conducted mainly in Europe (n=11), followed by America (n=5); Asia (n=3); and Africa (n=1). Using JBI’s critical appraisal checklist tool for prevalence studies, only 2 studies were considered at low risk of bias attaining a full score of 9; 5 studies attained a score 7-8; 8 studies a score 5-6, and 5 studies a score < 5 (**Supplementary S3**).

**Table 1:** Main characteristics of the studies included in the systematic review.

Study	Design	Setting	Country	Inclusion criteria	n. participants	Age, mean	Male, %	Follow-up, days, mean	History of pre-existing diabetes
Ayoubkhani 2021 (18)	Matched cohort, retrospective	Hospital	UK	Having a hospital episode with a primary diagnosis of Covid-19	47,780	64 (SD: 19.2)	54.9%	140 (SD: 50)	11.680 (24.4%)
Barret 2022 (19)	Matched cohort, retrospective	Community	US	i) aged <18 yrs; ii) acute infection of Covid-19	520,332	12.3 (SD: 4.3)	49.9%	> 30 days	0%
Basic-Jukic 2021 (20)	Cohort, prospective	Hospital	Croatia	i) Kidney-transplant patients with SARS-CoV2 infection; ii) two negative PCR	104	Median 56 (IQR: 45-65)	66.3%	Median 64: (IQR: 50-76)	21 (20.2%)
Basic-Jukic 2022 (21)	Cohort, retrospective	Hospital	Croatia	i) Kidney-transplant patients with SARS-CoV2 infection	308	Median 57 (IQR: 48-64)	64.9%	Median 126 (IQR: 114-159)	81 (26.3%)
Chafferdine 2021 (22)	Cross-sectional	Community	Tunisia	Diagnosis of Covid-19 in the past 2-24 weeks	798	49.9 (SD: 14.2)	39.5%	69 (SD: 3.1)	189 (23.7%)
Chowdhury 2021 (23)	Cohort, prospective	Community (80.2%), hospital (19.8%)	Bangladesh	PCR-confirmed SARS-Cov-2 cases	313	37.7 (SD: 13.7)	80.2%	140, max	20 (6,4%
Daugherty 2021 (24)	Cohort, retrospective	Community	US	i) age 18-65; ii) administrative claim with ICD-10 codeU07.1 or B34.2 or B97.29; iii) positive PCR test in outpatients laboratory dataset; iv) hospital admission with a diagnosis code of U07.1 or U07.2	266,586	41.7 (SD: 13.9)	47.6%	Median 95 (IQR: 42-135)	24.776 (9.3%)
Dennis 2021 (25)	Cohort, prospective	Community	UK	i) PCR-confirmed SARS-Cov-2 cases; ii) positive SARS_Cov-2 serology; iii) strong clinical suspicion	201	44 (SD: 11)	29%	Median 71 (IQR: 41-1149	2%
Dispinseri 2021 (26)	Cohort, retrospective	Hospital	Italy	i) PCR-confirmed SARS-Cov-2 infection and radiographic findings suggestive of Covid-19; ii) having a stored serum sample	150	64 (SD: 14)	69.3%	Median 202 (95%CI: 58-60)	22 (14.7%)
Legrand 2022 (27)	Cohort, retrospective	Hospital	France	i) adults; ii) positive SARS-Cov-2 PCR test; iii) available for 6-months follow-up	2,187	60.6 (SD: 14.8)	62.6%	180	431 (19.7)
Lewek 2021 (28)	Cross-sectional	Hospital	Poland	Convalescent PCR-confirmed Covid-19 patients with CVD complications	51	53 (SD: 16)	53%	60	NA
Maestre-Muniz 2021 (29)	Cross-sectional	Hospital	Spain	Adults with laboratory confirmed Covid-19 who attended to Emergency Room	543	NA	NA	12 months (SD: 1)	NA
Minstry 2021 (30)	Cohort, retrospective	Hospital	US	Confirmed Covid-19; ii) at least 1 glucose measurement before (>2 yrs) or after (<1 yr)	7,502	56 (SD: 19)	44%	NA	0%
Molinari 2021 (31)	Cohort, prospective	Hospital	Italy	Positive SARS-Cov-2 PCR testing for suspected infection; age >18 yrs; availability of serum sample	321	NA	NA	Median 213 (95%CI: 205-220)	56 (17%)
Montefusco 2021 (32)	Cohort, retrospective	Hospital	Italy	Admission to hospital with PCR-confirmed Covid-19	551	61 (SD: 0.7)	62%	6 months	86 (15.6%)
Nesan 2021 (33)	Cohort, prospective	Hospital	India	Admission to hospital with PCR-confirmed Covid-19	1,354	NA	73%	3 months	131 (9.7%)
Rezel-Potts 2022 (34)	Matched cohort, prospective	Community	UK	Covid-19 diagnosis	428,650	35 (22-50)	44%	12 months	0%
Xie 2022 (4)	Cohort, retrospective	Community	US	i) Having a positive SARS-Cov-2 test; ii) alive 30 days after positive test	181,280	57.7 (SD: 16.5)	86%	352 (244-406)	0%
Zhang 2022 (35)	Cohort, prospective	Hospital	China	i) PCR-confirmed Covid-19; ii) discharged from hospital	248	61 (54-68)	45%	12 months	25 (10.1%)
Zisis 2022 (36)	Cohort, retrospective	Community	US	PCR-confirmed Covid-19	50,450	55 (SD: 17)	40%	90	38,762 (76.8%)

SD, standard deviation; IQR, interquartile range; NA, not available.

### Demographics

The mean or median age of participants ranged from ~ 35 to 64 years, with the exception of one study that included children with a mean age of 12 years (19). The percentage of males ranged from ~ 29 to 80%. Most studies (65%) enrolled hospitalized patients, while the remaining were community-based studies. Follow-up after SARS-CoV-2 infection (considering both prevalent and incident diabetes) was quite variable, ranging from 2 to 12 months. Studies assessing the presence of diabetes within 60 days from SARS-CoV-2 infection were excluded leaving only studies with longer follow-up to examine the mid/long term effect of SARS-CoV-2 infection on metabolic impairment. This approach also limited the confounding effect of corticosteroid therapy in the acute or post-acute phase of COVID-19. Almost all the studies considered type 2 diabetes mellitus as the condition of interest, with only one study considering all type of diabetes. In this case, participants were aged < 18 years so type 1 diabetes mellitus was the most prevalent condition. Due to the difference in age population (children *vs* adults) and the condition mainly detected in this population (T1DM *vs* T2DM), such study (17) was excluded from pooled estimate calculation. Overall, 1.011.852 participants were included in the quantitative analysis.

### Prevalence of diabetes

**Figure 2:**
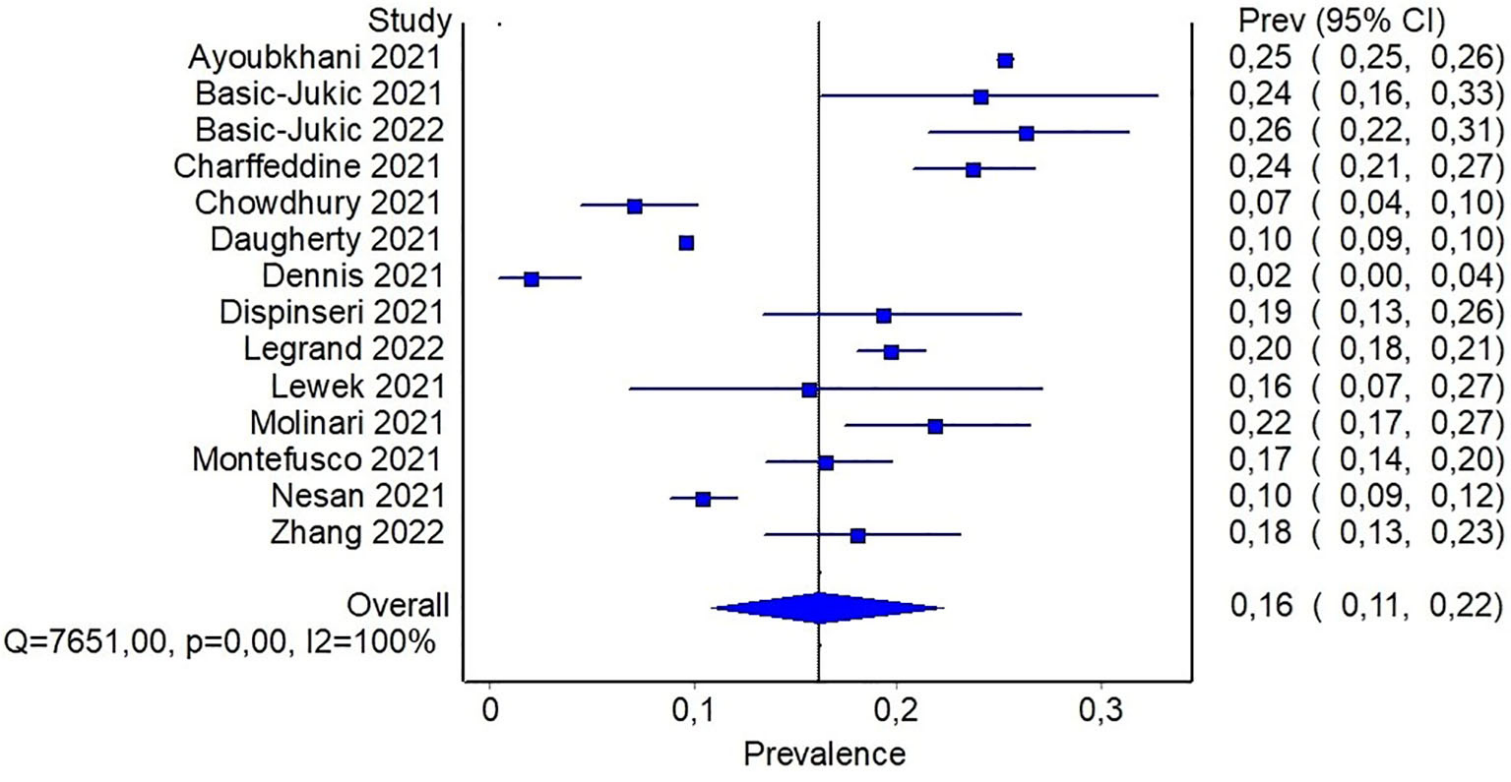
Pooled prevalence of diabetes in people with previous infection by SARS-CoV-2.

Data on diabetes prevalence was available in 14 studies. These studies were at intermediate/high risk of bias with only one study considered at low risk of bias. Methods of diabetes detection were quite variable, including registry codes, interviews by trained personnel, questionnaires, electronic medical records, or not specified in one case. Only one study defined diabetes with biochemical or clinical criteria, namely high FPG, HbA1c, or prescription of diabetes medication (26). In one study data about disease progression or poor glycemic control was available, involving ~ 35% of people with previous diabetes (23). In 8 studies diabetes prevalence was the primary outcome, while in the remaining studies it was considered as secondary outcome or reported as comorbidity. For the majority of the studies included in the quantitative analysis, no significant proportion of lost to follow-up or died was reported (<5%); in 3 studies this proportion was higher ranging from 11 to 19%. Reported prevalence estimates were quite variable and ranged from a minimum of 2% in a community-based study to a maximum of 26% in patients with renal transplant. A combined total of 320,948 participants across the studies reporting on diabetes prevalence were pooled with 38,731 cases of diabetes. After fitting a random effect model to the fourteen representative studies, the pooled prevalence estimate for total cases of diabetes was 16% (95%CI: 11-22%), as shown in **Figure 2**.

**Figure 3:**
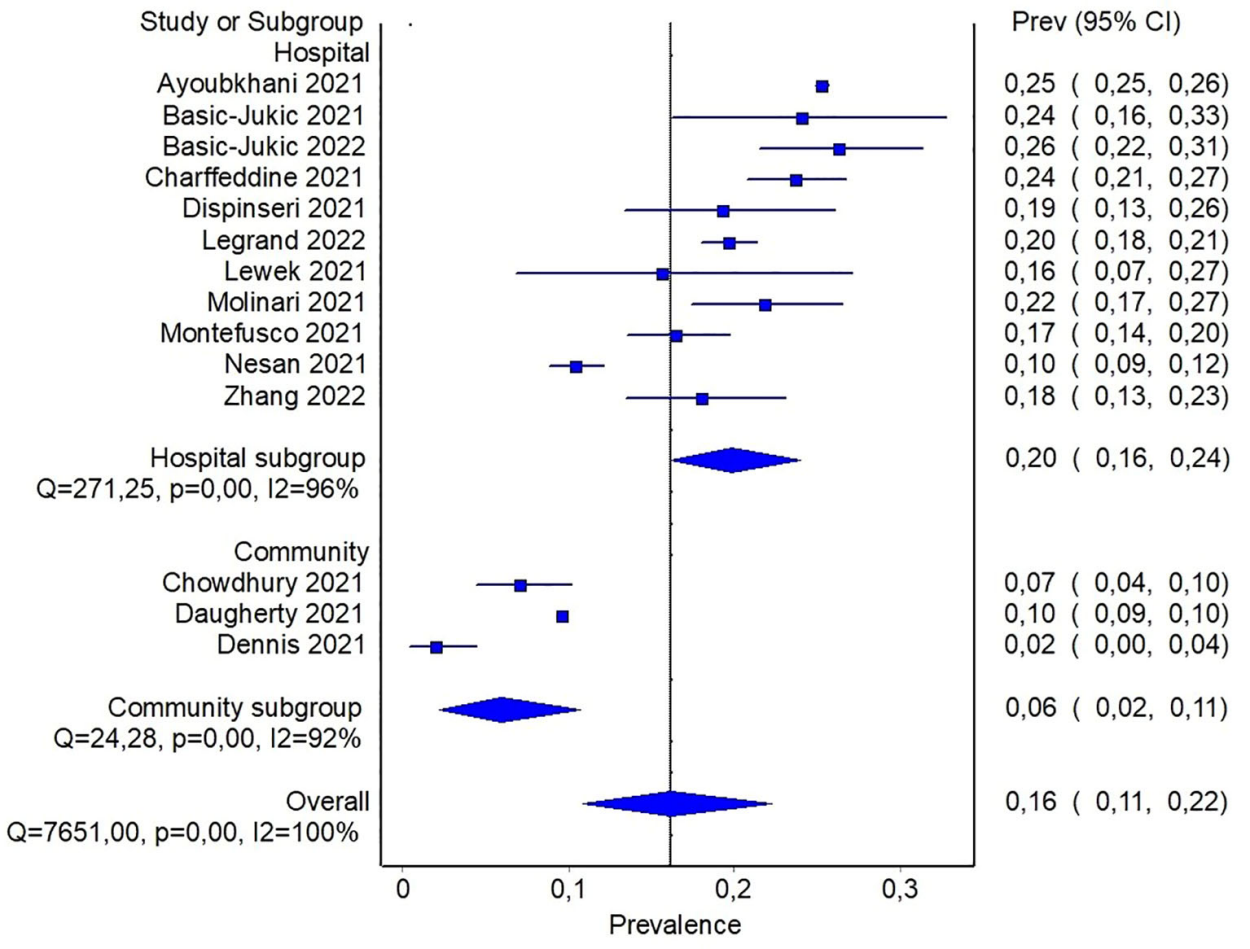
Pooled prevalence of diabetes in patients with previous hospitalization for COVID-19 or from community.

In order to detect potential sources of heterogeneity among the studies, a subgroup analysis based on the severity of previous COVID-19 was carried out. It was assumed that inpatients recruited from hospital records presented a more severe disease than individuals recruited in community-based studies. In this subgroup analysis, the pooled prevalence of diabetes was 6% (95%CI: 2-11%) when community-based studies were considered, and reach 20% (95%CI: 16-24%) for studies including only patients with previous hospitalization (**Figure 3**). In the latter analysis, it should be noticed that only three studies reported data about community participants, and all of them included also a small proportion of participants with previous hospitalization, ranging from ~ 8% to 19%. In order to detect other sources of heterogeneity, a random effect model including patients stratified by age was fitted. Specifically, combined prevalence of diabetes in participants aged < 60 was 14% (95%CI: 10-19%), while the one from studies including patients aged > 60 years was slightly higher, namely 21% (95%CI: 17-25%) (**Supplementary S5**).

### Proportion of incident cases diabetes

Fifteen studies reported the number of new onset type 2 diabetes cases after SARS-CoV-2 infection. About half of them (46%) were considered to be at intermediate/low risk of bias. Again, high variability regarding diabetes definition exists among the studies. In three studies, a matched-cohort study design was used to compare diabetes incidence in a group of participants with previous SARS-CoV-2 infection with a matched control group with no previous infection. In such cases people with prevalent diabetes were specifically excluded at study entry (4, 30, 34). These studies showed that in the cohort of people with previous SARS-CoV-2 infection the incidence of diabetes was higher than the corresponding historical or contemporary non-COVID-19 control cohort, suggesting that infected people might be at higher risk of developing diabetes than people who was not infected. Similar findings were reported by Barret et al. who demonstrated that type 1 diabetes incidence was higher in a pediatric cohort of patients with COVID-19 than in matched cohorts of participants without COVID-19, or with other respiratory acute infection, or without both of them (19).

**Figure 4:**
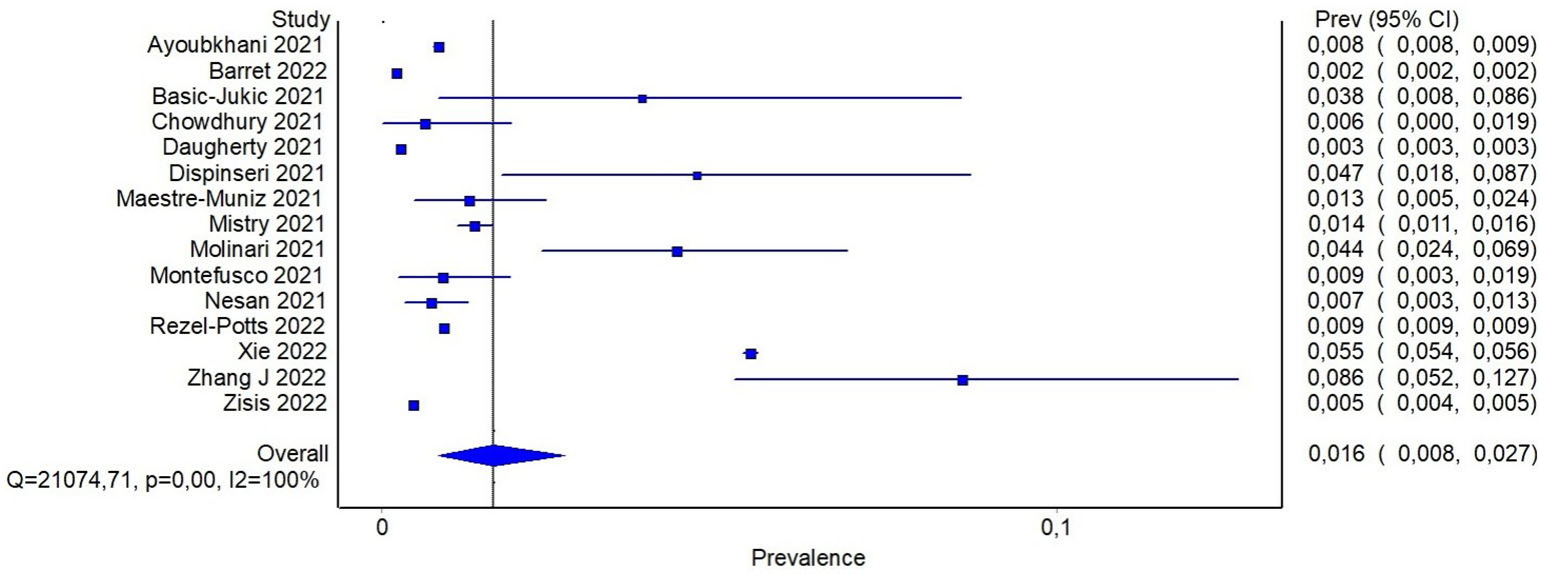
Pooled proportion of new onset diabetes after SARS-CoV-2 infection.

**Figure 5:**
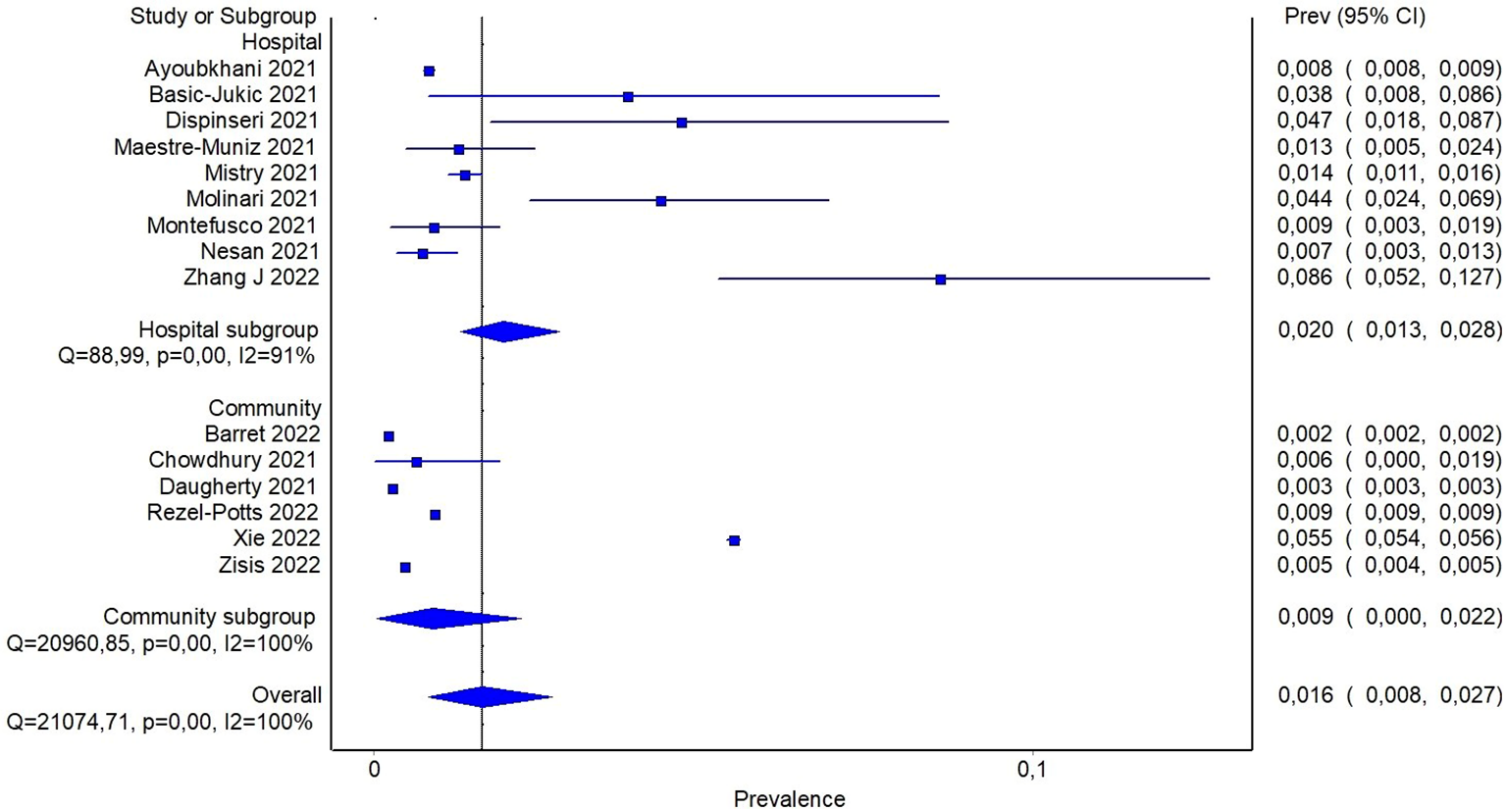
Pooled proportion of new onset diabetes in patients with previous hospitalization for COVID-19 or from community.

Reported proportion estimate of new-onset diabetes diagnosis ranged from 0.2% to 8.6%. In the random effect model, 1,506,137 participants across the studies were pooled with 16,502 cases of new-onset diabetes. The pooled proportion of new onset diagnosis after at least 60 days from SARS-CoV-2 infection was 1.6% (95%CI: 0.8-2.7%) (**Figure 4**). To further investigate whether the severity of COVID-19 may explain, at least in part, the observed heterogeneity among the studies, a subgroup analysis based on previous hospitalization for COVID-19 was performed. Specifically, the pooled estimate of new-onset diabetes proportion in patients with previous hospital admission for COVID-19 was 2% (95%CI: 1.3-2.8%), while in participants from community it was 0.9% (95%CI: 0.04-2.2%) (**Figure 5**). If compared with the corresponding subgroup analysis for total diabetes prevalence, new-onset diagnosis proportion seems influenced by previous hospitalization to a lesser extent. Subgroup analysis based on age of participants showed that in patients aged > 60 years the proportion of new-onset diabetes is slightly higher than in younger patients (2.8% [95%CI: 1.1-4.9%] and 1.1% [95%CI: 0.3-2.3], respectively) (**Supplementary S5**).

### Quality of the body of evidence

While no formal guidance for using GRADE for assessment of evidence in systematic reviews of prevalence, there is some guidance into approaching this issue using the GRADE for prognosis (37). With respect to risk of bias, the main concerns are about the methods used for the identification of the condition (Question 6 of JBI’s checklist) and the low response rate (Question 9 of JBI’s checklist), which reached the lowest score. Overall, sensitivity analysis demonstrated that the inclusion of studies at high risk of bias did not change significantly the results, so they were included in the full analysis (**Supplementary S4**). Analogous findings emerged with the leave-one-out method (**Supplementary S7**). Inconsistency was evaluated observing the variability of point estimates and the extent of overlap in confidence interval. Overall, inconsistency was judged high for both prevalence of diabetes and proportion of new cases of diabetes. At this regard, subgroup analysis tried to explain part of heterogeneity with the severity of previous COVID-19, especially when considering prevalence of diabetes as condition of interest.

Indirectness was evaluated considering the overlap between the population of interest and the studied populations. No specific source of indirectness was detected in the body of evidence since almost all the studies included participants with PCR-confirmed SARS-CoV-2 infection. Finally, asymmetry was detected in funnel plot, suggesting the presence of significant publication bias (**Supplementary S6**).

## Discussion

In this systematic review we report the prevalence of diabetes in people with previous SARS-CoV-2 infection. Moreover, we describe the proportion on incident cases of diabetes in this population. The prevalence of diabetes was higher in patients who were hospitalized for COVID-19 and who were older than 60 years old in comparison to people not hospitalized and younger. It seems that the proportion of incident cases of diabetes may be less influenced by previous hospitalization than prevalence, suggesting that the relationship between COVID-19 and diabetes development may be less influenced by severity of the disease.

The association between SARS-CoV-2 infection and the risk of developing diabetes after COVID-19 is poorly understood. It is plausible that the direct attack of pancreatic β-cells by the virus may lead to altered glucose metabolism and diabetes in healthy people. In addition, other mechanisms have been proposed, such as autonomic dysfunction, stress hyperglycemia induced by cytokine storm, and low-grade inflammation leading to insulin resistance (38). In this systematic review we showed that prevalence of diabetes in people with previous SARS-CoV-2 infection is ~16%, and it reaches 20% when individuals who had experienced severe forms of COVID-19 were considered. To contextualize this data, it can be noticed that the prevalence of diabetes in the general population has been estimated ranging from 9% and 16% approximately, depending on geographical area (39). As already mentioned, diabetes is frequent in patients with COVID-19 and it represents a significant risk factor for severe COVID-19 (40), so it is reasonable that people with a history of severe COVID-19 may have diabetes more frequently than people not infected. Therefore, the proportion of COVID-19 survivors who had, or have developed, diabetes may be higher than expected in the general population.

A large body of evidence supports the hypothesis that COVID-19 may increase the risk of incident diabetes. For example, in a recent systematic review the risk of new-onset diabetes after COVID-19 was reported higher than in non-COVID-19 population, ranging from 11% to 276% (41). This systematic review includes three matched-cohort studies that reported increased risk of diabetes after SARS-CoV-2 infection in comparison with historical and not-historical control cohorts (4, 19, 34).

Debate regarding the association between COVID-19 severity and diabetes development is also reported. For example, in the study of Holman et al. the incidence of diabetes was not significantly different between patients with COVID-19 and pneumonia in comparison to patients with COVID-19 without pneumonia, irrespective of hospitalization (42). In the systematic review of Almas et al, the pooled prevalence of new onset diabetes was 0.46%, slightly lower than the one observed in the present review (43). It should be noticed that in this case only three of the included studies reported about new onset diabetes, with a significantly lower number of patients, leading to a higher grade of imprecision of that estimate. Satish et al. reported a pooled proportion of new onset diabetes of ~ 14% in individuals with COVID-19 (44). In that study diabetes diagnosis was made during hospitalization, or often not specified, including also the cases of secondary hyperglycemia due to infection itself and/or corticosteroid therapy. Several other meta-analyses have been published on the association between diabetes and COVID-19. Most of them have focused on the new-onset diabetes after COVID-19, reporting significant increased risk in COVID-19 patients compared with non-COVID-19 controls. However, all these studies considered follow-up periods that were too close to the detection of infection - thus not resolving the issue of potential confounding by stress hyperglycemia – and did not distinguish new onset of diabetes according to COVID-19 severity (45–48).

Another systematic review estimated an incidence of diabetes of 2.19% in studies with a follow-up of less than three months, and 0.91% in studies with a follow-up of more than six months, providing results that are not dissimilar to ours in terms of new cases of diabetes after COVID-19 (49). Finally, an extensive meta-analysis of 729 studies quantified the overall prevalence of diabetes in various COVID-19 disease stages, with an overall estimate of 14% which is quite close to what was observed in our study. However, it did not provide data on the incidence of new cases of diabetes after COVID-19 (50).

The strength of the present systematic review is that our results are free from the confounding effect of stress-induced hyperglycemia activated by the infection and corticosteroid therapy, frequently used in these cases. Additionally, we focused on both incidence and prevalence data in the attempt to provide a comprehensive view of the relationship between COVID-19 and diabetes. With respect to the limitations of the body of evidence collected in this systematic review, only 2 studies out of 20 were considered at low risk of bias and only English language papers were included. However, we believe the impact of language bias was somewhat limited since the vast majority of literature in this field is in English. In addition, non-English language reports accounted for just 1.5% of the total reports searched. Second, an accurate measure of incidence of diabetes was not available from the included studies, but only the proportion of incident cases in studies considering different follow-up, ranging from 2 to 12 months. We should also acknowledge that, while a follow-up period of at least two months after SARS-CoV-2 infection may be enough to minimize the hyperglycemic effect of concomitant corticosteroid therapy, the confounding effect of persistent inflammation - particularly after the most severe form of COVID-19 (51, 52) - on insulin-resistance and glucose homeostasis cannot be ruled out. The consequence is that the generalization of these results should be considered with caution.

In conclusion, this systematic review shows that diabetes is common in individuals who have experienced SARS-CoV-2 infection, especially if they had required hospitalization. This information may be helpful for clinicians and health care systems to plan adequate screening and follow-up programs in this specific population.

## Data availability statement

The original contributions presented in the study are included in the article/**Supplementary Materials**, further inquiries can be directed to the corresponding author.

## Author contributions

CB and AA contributed equally to this manuscript. CB, AA, AB contributed to conception and design of the study. CB, ID, SB, LS organized the database. CB performed the statistical analysis. CB and AA wrote the first draft of the manuscript. ID, SB, MM, LS wrote sections of the manuscript. AB and DL contributed to manuscript revision. All authors contributed to the article and approved the submitted version.

## References

[1] Harding JL Ali MK Gander JC Patzer RE . 174-LB: diabetes as a risk factor for long-COVID-19—a scoping review. Diabetes (2022) 71:174–LB. doi: 10.2337/db22-174-LB

[2] Onder G Rezza G Brusaferro S . Case-fatality rate and characteristics of patients dying in relation to COVID-19 in Italy. JAMA (2020) 323:1775–6. doi: 10.1001/jama.2020.4683 32203977

[3] Muniyappa R Gubbi S . COVID-19 pandemic, coronaviruses, and diabetes mellitus. Am J Physiol Endocrinol Metab (2020) 318:E736–e741. doi: 10.1152/ajpendo.00124.2020 32228322 PMC7191633

[4] Xie Y Al-Aly Z . Risks and burdens of incident diabetes in long COVID: a cohort study. Lancet Diabetes Endocrinol (2022) 10:311–21. doi: 10.1016/S2213-8587(22)00044-4 PMC8937253 35325624

[5] Ren H Yang Y Wang F Yan Y Shi X Dong K . Association of the insulin resistance marker TyG index with the severity and mortality of COVID-19. Cardiovasc Diabetol (2020) 19:58. doi: 10.1186/s12933-020-01035-2 32393351 PMC7213552

[6] Chee YJ Ng SJH Yeoh E . Diabetic ketoacidosis precipitated by Covid-19 in a patient with newly diagnosed diabetes mellitus. Diabetes Res Clin Pract (2020) 164:108166. doi: 10.1016/j.diabres.2020.108166 32339533 PMC7194589

[7] Lan J Ge J Yu J Shan S Zhou H Fan S . Structure of the SARS-CoV-2 spike receptor-binding domain bound to the ACE2 receptor. Nature (2020) 581:215–20. doi: 10.1038/s41586-020-2180-5 32225176

[8] Shirbhate E Pandey J Patel VK Kamal M Jawaid T Gorain B . Understanding the role of ACE-2 receptor in pathogenesis of COVID-19 disease: a potential approach for therapeutic intervention. Pharmacol Rep (2021) 73:1539–50. doi: 10.1007/s43440-021-00303-6 PMC8236094 34176080

[9] Steenblock C Schwarz PEH Ludwig B Linkermann A Zimmet P Kulebyakin K . COVID-19 and metabolic disease: mechanisms and clinical management. Lancet Diabetes Endocrinol (2021) 9:786–98. doi: 10.1016/S2213-8587(21)00244-8 PMC8489878 34619105

[10] Rathmann W Kuss O Kostev K . Incidence of newly diagnosed diabetes after Covid-19. Diabetologia (2022) 65:949–54. doi: 10.1007/s00125-022-05670-0 PMC8923743 35292829

[11] Virgilio E Trevisan C Abbatecola A Malara A Palmieri A Fedele G . Diabetes affects antibody response to SARS-coV-2 vaccination in older residents of long-term care facilities: data from the geroCovid vax study. Diabetes Care (2022) 45:2935–42. doi: 10.2337/dc22-1255 36201657

[12] Page MJ McKenzie JE Bossuyt PM Boutron I Hoffmann TC Mulrow CD . The PRISMA 2020 statement: an updated guideline for reporting systematic reviews. BMJ (2021) 372:n71. doi: 10.1136/bmj.n71 33782057 PMC8005924

[13] Munn Z Moola S Lisy K Riitano D Tufanaru C . Methodological guidance for systematic reviews of observational epidemiological studies reporting prevalence and cumulative incidence data. Int J Evid Based Healthc (2015) 13:147–53. doi: 10.1097/XEB.0000000000000054 26317388

[14] Naing L Winn T Rusli B . Practical issues in calculating the sample size for prevalence studies. Arch Orofacial Sci (2006) 1:6.

[15] Barendregt JJ Doi SA Lee YY Norman RE Vos T . Meta-analysis of prevalence. J Epidemiol Community Health (2013) 67:974–8. doi: 10.1136/jech-2013-203104 23963506

[16] Estiri H Strasser ZH Brat GA Semenov YR; Consortium for Characterization of COVID-19 by EHR (4CE), Patel CJ . Evolving phenotypes of non-hospitalized patients that indicate long COVID. BMC Med (2021) 19:249. doi: 10.1186/s12916-021-02115-0 34565368 PMC8474909

[17] Zhang HG Dagliati A Shakeri Hossein Abad Z Xiong X Bonzel CL Xia Z . International electronic health record-derived post-acute sequelae profiles of COVID-19 patients. NPJ Digit Med (2022) 5:81. doi: 10.1038/s41746-022-00623-8 35768548 PMC9242995

[18] Ayoubkhani D Khunti K Nafilyan V Maddox T Humberstone B Diamond I . Post-covid syndrome in individuals admitted to hospital with covid-19: retrospective cohort study. BMJ (Clinical Res ed) (2021) 372:n693. doi: 10.1136/bmj.n693 PMC8010267 33789877

[19] Barrett CE Koyama AK Alvarez P Chow W Lundeen EA Perrine CG . Risk for newly diagnosed diabetes >30 days after SARS-coV-2 infection among persons aged <18 years - United States, march 1, 2020-june 28, 2021. MMWR Morbidity mortality weekly Rep (2022) 71:59–65. doi: 10.15585/mmwr.mm7102e2 PMC8757617 35025851

[20] Basic-Jukic N Racki S Tolj I Aleckovic M Babovic B Juric I . Hospitalization and death after recovery from acute COVID-19 among renal transplant recipients. Clin Transplant (2022) 36(4):e14572. doi: 10.1111/ctr.14572 34967958

[21] Basic-Jukic N Juric I Furic-Cunko V Katalinic L Radic J Bosnjak Z . Follow-up of renal transplant recipients after acute COVID-19-A prospective cohort single-center study. Immunity Inflammation Dis (2021) 9:1563–72. doi: 10.1002/iid3.509 PMC8426882 34414665

[22] Charfeddine S Ibn Hadj Amor H Jdidi J Torjmen S Kraiem S Hammami R . Long COVID 19 syndrome: is it related to microcirculation and endothelial dysfunction? Insights from TUN-endCOV study. Front Cardiovasc Med (2021) 8:745758. doi: 10.3389/fcvm.2021.745758 34917659 PMC8670225

[23] Chowdhury ATMM Karim R Ali A Islam J Li Y He S . Clinical characteristics and the long-term post-recovery manifestations of the COVID-19 patients-A prospective multicenter cross-sectional study. Front Med (2021) 8:663670. doi: 10.3389/fmed.2021.663670 PMC8416537 34490284

[24] Daugherty SE Guo Y Heath K Dasmariñas MC Jubilo KG Samranvedhya J . Risk of clinical sequelae after the acute phase of SARS-CoV-2 infection: retrospective cohort study. BMJ (2021) 373:n1098. doi: 10.1136/bmj.n1098 34011492 PMC8132065

[25] Dennis A Wamil M Alberts J Oben J Cuthbertson DJ Wootton D . Multiorgan impairment in low-risk individuals with post-COVID-19 syndrome: a prospective, community-based study. BMJ Open (2021) 11(3):e048391. doi: 10.1136/bmjopen-2020-048391 PMC8727683 33785495

[26] Dispinseri S Lampasona V Secchi M Cara A Bazzigaluppi E Negri D . Robust neutralizing antibodies to SARS-coV-2 develop and persist in subjects with diabetes and COVID-19 pneumonia. J Clin Endocrinol Metab (2021) 106:1472–81. doi: 10.1210/clinem/dgab055 PMC7928901 33513242

[27] Legrand M Fong N Laouénan C Ghosn J Thill B Faure K . Risk factors of long term symptoms and outcomes among patients discharged after covid-19: prospective, multicentre observational study. BMJ Med (2022) 1:e000093. doi: 10.1136/bmjmed-2021-000093 PMC9951375 36936553

[28] Lewek J Jatczak-Pawlik I Maciejewski M Jankowski P Banach M . COVID-19 and cardiovascular complications - preliminary results of the LATE-COVID study. Arch Med science: AMS (2021) 17:818–22. doi: 10.5114/aoms/134211 PMC8130484 34025853

[29] Maestre-Muñiz MM Arias Á Mata-Vázquez E Martín-Toledano M López-Larramona G Ruiz-Chicote AM . Long-Term Outcomes of Patients with Coronavirus Disease 2019 at One Year after Hospital Discharge. J Clin Med (2021) 10(13):2945. doi: 10.3390/jcm10132945 34209085 PMC8269002

[30] Mistry S Gouripeddi R Facelli JC . Data-driven identification of temporal glucose patterns in a large cohort of nondiabetic patients with COVID-19 using time-series clustering. JAMA Open (2021) 4:ooab063. doi: 10.1093/jamiaopen/ooab063 PMC8364667 34409266

[31] Molinari C Laurenzi A Caretto A Rovere-Querini P Ciceri F . Dysglycemia after COVID-19 pneumonia: a six-month cohort study. Acta diabetol (2021) 58:1481–90. doi: 10.1007/s00592-021-01751-5 PMC8177035 34089096

[32] Montefusco L Ben Nasr M D’Addio F Loretelli C Rossi A Pastore I . Acute and long-term disruption of glycometabolic control after SARS-CoV-2 infection. Nat Metab (2021) 3:774–85. doi: 10.1038/s42255-021-00407-6 PMC9931026 34035524

[33] Nesan GSCQ Keerthana D Yamini R Jain T Kumar D Eashwer A . 3-month symptom-based ambidirectional follow-up study among recovered COVID-19 patients from a tertiary care hospital using telehealth in chennai, India. Inquiry: J Med Care organizat provision financing 58:469580211060165. doi: 10.1177/00469580211060165 PMC8721686 34915771

[34] Rezel-Potts E Douiri A Sun X Chowienczyk PJ Shah AM Gulliford MC . Cardiometabolic outcomes up to 12 months after COVID-19 infection. A matched cohort study in the UK. PloS Med (2022) 19:e1004052. doi: 10.1371/journal.pmed.1004052 35853019 PMC9295991

[35] Zhang J Shu T Zhu R Yang F Zhang B Lai X . The long-term effect of COVID-19 disease severity on risk of diabetes incidence and the near 1-year follow-up outcomes among postdischarge patients in wuhan. J Clin Med (2022) 11(11):3094. doi: 10.3390/jcm11113094 35683480 PMC9181214

[36] Zisis SN Durieux JC Mouchati C Perez JA McComsey GA . The protective effect of coronavirus disease 2019 (COVID-19) vaccination on postacute sequelae of COVID-19: A multicenter study from a large national health research network. Open Forum Infect Dis (2022) 9:ofac228. doi: 10.1093/ofid/ofac228 35818362 PMC9129153

[37] Iorio A Spencer FA Falavigna M Alba C Lang E Burnand B . Use of GRADE for assessment of evidence about prognosis: rating confidence in estimates of event rates in broad categories of patients. BMJ (2015) 350:h870. doi: 10.1136/bmj.h870 25775931

[38] Kazakou P Lambadiari V Ikonomidis I Kountouri A Panagopoulos G Athanasopoulos S . Diabetes and COVID-19; A bidirectional interplay. Front Endocrinol (Lausanne) (2022) 13:780663. doi: 10.3389/fendo.2022.780663 35250853 PMC8891603

[39] International diabetes federation. Available at: https://diabetesatlas.org/data/en/ (Accessed 20 January 2023).

[40] Pellicori P Doolub G Wong CM Lee KS Mangion K Ahmad M . COVID-19 and its cardiovascular effects: a systematic review of prevalence studies. Cochrane Database Syst Rev (2021) 3:Cd013879. doi: 10.1002/14651858.CD013879 33704775 PMC8078349

[41] Harding JL Oviedo SA Ali MK Ofotokun I Gander JC Patel SA . The bidirectional association between diabetes and long-COVID-19 - A systematic review. Diabetes Res Clin Pract (2022) 195:110202. doi: 10.1016/j.diabres.2022.110202 36496030 PMC9727969

[42] Holman N Barron E Young B Gregg EW Khunti K Valabhji J . Comparative incidence of diabetes following hospital admission for COVID-19 and pneumonia: A cohort study. Diabetes Care (2023) 46:1–6. doi: 10.2337/dc22-0670 36657086

[43] Almas T Malik J Alsubai AK Jawad Zaidi SM Iqbal R Khan K . Post-acute COVID-19 syndrome and its prolonged effects: an updated systematic review. Ann Med Surg (Lond) (2022) 80:103995. doi: 10.1016/j.amsu.2022.103995 35721785 PMC9197790

[44] Sathish T Kapoor N Cao Y Tapp RJ Zimmet P . Proportion of newly diagnosed diabetes in COVID-19 patients: a systematic review and meta-analysis. Diabetes Obes Metab (2021) 23:870–4. doi: 10.1111/dom.14269 PMC7753574 33245182

[45] Lai H Yang M Sun M Pan B Wang Q Wang J . Risk of incident diabetes after COVID-19 infection: a systematic review and meta-analysis. Metabolism (2022) 137:155330. doi: 10.1016/j.metabol.2022.155330 36220361 PMC9546784

[46] Banerjee M Pal R Dutta S . Risk of incident diabetes post-COVID-19: a systematic review and meta-analysis. Prim Care Diabetes. (2022) 16:591–3. doi: 10.1016/j.pcd.2022.05.009 PMC9148988 35654679

[47] Wrona M Skrypnik D . New-onset diabetes mellitus, hypertension, dyslipidaemia as sequelae of COVID-19 infection-systematic review. Int J Environ Res Public Health (2022) 19:13280. doi: 10.3390/ijerph192013280 36293857 PMC9602450

[48] Ssentongo P Zhang Y Witmer L Chinchilli VM Ba DM . Association of COVID-19 with diabetes: a systematic review and meta-analysis. Sci Rep (2022) 12:20191. doi: 10.1038/s41598-022-24185-7 36418912 PMC9684130

[49] Zhang T Mei Q Zhang Z Walline JH Liu Y Zhu H . Risk for newly diagnosed diabetes after COVID-19: a systematic review and meta-analysis. BMC Med (2022) 20:444. doi: 10.1186/s12916-022-02656-y 36380329 PMC9666960

[50] Li R Shen M Yang Q Fairley CK Chai Z McIntyre R . Global diabetes prevalence in COVID-19 patients and contribution to COVID-19- related severity and mortality: A systematic review and meta-analysis. Diabetes Care (2023) 46:890–7. doi: 10.2337/dc22-1943 PMC10090902 36826982

[51] Gameil MA Marzouk RE Elsebaie AH Rozaik SE . Long-term clinical and biochemical residue after COVID-19 recovery. Egypt Liver J (2021) 11:74. doi: 10.1186/s43066-021-00144-1 34777873 PMC8435147

[52] Mandal S Barnett J Brill SE Brown JS Denneny EK Hare SS . ‘Long-COVID’: a cross-sectional study of persisting symptoms, biomarker and imaging abnorMalities following hospitalisation for COVID-19. Thorax (2021) 76:396–8. doi: 10.1136/thoraxjnl-2020-215818 PMC7615158 33172844

