# Supplementary material for: Prevalence and risk of new-onset diabetes mellitus after COVID-19: a systematic review and meta-analysis
Source: Front Endocrinol (Lausanne). 2023 Sep 4;14:1215879. doi: 10.3389/fendo.2023.1215879 (PMC10507325; doi:10.3389/fendo.2023.1215879)
Supplement: Supplementary file 1 [file DataSheet_1.zip › Supplementary S2.docx]

**Supplementary S2.** Full literature search strategy used for MEDLINE (A); Web of Science (B); Scopus (C); and clinicaltrials.gov (D). MEDLINE was searched using both the Advanced Search and the Medical Subjects Headings (MeSH) Search.

**(A) MEDLINE**

1. Diabet*.ti,ab.

2. “diabetes mellitus” .ti,ab.

3. “non-insulin dependent diabetes” .ti,ab.

4. “insulin dependent diabetes”.ti,ab.

5. NIDDM.ti,ab.

6. IDDM.ti,ab.

7. T2D.ti,ab.

8. T1D.ti,ab.

9. “long covid”.ti,ab.

10. "long COVID syndrome”.ti,ab.

11. "long COVID symptoms".ti,ab.

12. "long haul COVID".ti,ab.

13. "long hauler COVID".ti,ab.

14. "chronic COVID syndrome".ti,ab.

15. PACS.ti,ab.

16. “Post-acute coronavirus disease 2019 syndrome”.ti,ab.

17. "chronic COVID symptoms".ti,ab.

18. "post-acute COVID syndrome".ti,ab.

19. "post-acute COVID symptoms".ti,ab.

20. "persistent COVID syndrome".ti,ab.

21. "post-COVID".ti,ab.

22. "COVID sequelae".ti,ab.

23. "persistent COVID symptoms".ti,ab.

24. OR/1-8

25. OR/1-23

26. 24 AND 25

Medical Subjects Headings - MeSH Search

("post-acute COVID-19 syndrome" [Supplementary Concept]) AND "Diabetes Mellitus"[Majr]

**(B) Web of Science**

**Diabet* OR “diabetes mellitus” OR “non-insulin dependent diabetes” OR “insulin dependent diabetes” OR NIDDM OR IDDM OR T2D OR T1D** (Abstract)

AND **“long covid” OR "long COVID syndrome” OR "long COVID symptoms" OR "long haul COVID" OR "long hauler COVID" OR "chronic COVID syndrome" OR PACS OR “Post-acute coronavirus disease 2019 syndrome” OR "chronic COVID symptoms" OR "post-acute COVID syndrome" OR "post-acute COVID symptoms" OR "persistent COVID syndrome" OR "post-COVID" OR "COVID sequalae" OR "persistent COVID symptoms"** (Abstract)

**(C) Scopus**

TITLE-ABS ( diabet* OR "diabetes mellitus" OR "non-insulin dependent diabetes" OR "insulin dependent diabetes" OR niddm OR iddm OR t2d OR t1d AND "long covid" OR "long COVID syndrome" OR "long COVID symptoms" OR "long haul COVID" OR "long hauler COVID" OR "chronic COVID syndrome" OR pacs OR "Post-acute coronavirus disease 2019 syndrome" OR "chronic COVID symptoms" OR "post-acute COVID syndrome" OR "post-acute COVID symptoms" OR "persistent COVID syndrome" OR "post-COVID" OR "COVID sequalae" OR "persistent COVID symptoms" )

**(D) ClinicalTrials.gov**

Diabet* OR “diabetes mellitus” OR “non-insulin dependent diabetes” OR “insulin dependent diabetes” OR NIDDM OR IDDM OR T2D OR T1D | “long covid” OR "long COVID syndrome” OR "long COVID symptoms" OR "long haul COVID" OR "long hauler COVID" OR "chronic COVID syndrome" OR PACS OR “Post-acute coronavirus disease 2019 syndrome” OR "chronic COVID symptoms" OR "post-acute COVID syndrome | Adult, Older Adult
