# Supplementary material for: Prevalence and risk of new-onset diabetes mellitus after COVID-19: a systematic review and meta-analysis
Source: Front Endocrinol (Lausanne). 2023 Sep 4;14:1215879. doi: 10.3389/fendo.2023.1215879 (PMC10507325; doi:10.3389/fendo.2023.1215879)
Supplement: Supplementary file 1 [file DataSheet_1.zip › Supplementary S3.docx]

| **Studi ID** | **Score for critical appraisal** |
| --- | --- |
| Ayoubkhani 2021 | 9 |
| Barret 2022 | 9 |
| Basic-Jukic 2021 | 1 |
| Basic-Jukic 2022 | 3 |
| Charffeddine 2021 | 5 |
| Chowdhury 2021 | 6 |
| Daugherty 2021 | 6 |
| Dennis 2021 | 1 |
| Dispinseri 2021 | 5 |
| Lewek 2021 | 4 |
| Maestre-Muniz 2021 | 7 |
| Mistry 2021 | 6 |
| Molinari 2021 | 5 |
| Montefusco 2021 | 7 |
| Nesan 2021 | 4 |
| Legrand 2022 | 6 |
| Rezel-Potts 2022 | 8 |
| Xie 2022 | 8 |
| Zhang 2022 | 5 |
| Zsis 2022 | 7 |

**Supplementary S3.** Critical appraisal for the selected studies assessed by the Joanna Briggs Institute (JBI) checklist for prevalence studies. The checklist includes 9 questions; for each question, a score is assigned basing on the following answer: yes = 1 point; no = 0 point; unclear = 0 point.
