# Supplementary material for: Prevalence and risk of new-onset diabetes mellitus after COVID-19: a systematic review and meta-analysis
Source: Front Endocrinol (Lausanne). 2023 Sep 4;14:1215879. doi: 10.3389/fendo.2023.1215879 (PMC10507325; doi:10.3389/fendo.2023.1215879)
Supplement: Supplementary file 1 [file DataSheet_1.zip › Supplementary S7.docx]

**Supplementary S7** Sensitivity analysis showing pooled estimates for prevalence of diabetes (A) and proportion of new-onset diabetes (B) using the leave-one-out method.

(A)

| **Excluded study** | **Leave-one-out results** | |
| --- | --- | --- |
|  | **Pooled Prevalence** | **95%CI** |
| Basic-Jukic 2022 | 0.155 | 0.101-0.217 |
| Ayoubkhani 2021 | 0.156 | 0.118-0.198 |
| Basic-Jukic 2021 | 0.156 | 0.102-0.219 |
| Chaffeddine 2021 | 0.156 | 0.102-0.219 |
| Molinari 2021 | 0.157 | 0.103-0.221 |
| Dispinseri 2021 | 0.159 | 0.105-0.223 |
| Legrand 2022 | 0.159 | 0.104-0.223 |
| Zhang 2022 | 0.160 | 0.105-0.224 |
| Montefusco 2021 | 0.161 | 0.106-0.225 |
| Lewek 2021 | 0.162 | 0.107-0.225 |
| Nesan 2021 | 0.167 | 0.110-0.232 |
| Daugherty 2021 | 0.169 | 0.130-0.212 |
| Chowdhury 2021 | 0.170 | 0.114-0.235 |
| Dennis 2021 | 0.177 | 0.120-0.242 |

(B)

| **Excluded study** | **Leave-one-out results** | |
| --- | --- | --- |
|  | **Pooled Prevalence** | **95%CI** |
| Xie 2022 | 0.011 | 0.008-0.015 |
| Zhang J 2022 | 0.014 | 0.006-0.024 |
| Basic-Jukic 2021 | 0.015 | 0.007-0.026 |
| Dispinseri 2021 | 0.015 | 0.007-0.026 |
| Molinari 2021 | 0.015 | 0.007-0.026 |
| Ayoubkhani 2021 | 0.017 | 0.008-0.029 |
| Chowdhury 2021 | 0.017 | 0.009-0.028 |
| Maestre-Muniz 2021 | 0.017 | 0.008-0.028 |
| Minstry 2021 | 0.017 | 0.008-0.028 |
| Montefusco 2021 | 0.017 | 0.008-0.028 |
| Nesan 2021 | 0.017 | 0.009-0.029 |
| Rezel-Potts 2022 | 0.017 | 0.007-0.032 |
| Barrett 2022 | 0.018 | 0.009-0.031 |
| Daugherty 2021 | 0.018 | 0.008-0.031 |
| Zisis 2022 | 0.018 | 0.009-0.030 |
