# Supplementary figures and images for: Prevalence and risk of new-onset diabetes mellitus after COVID-19: a systematic review and meta-analysis
Source: Front Endocrinol (Lausanne). 2023 Sep 4;14:1215879. doi: 10.3389/fendo.2023.1215879 (PMC10507325; doi:10.3389/fendo.2023.1215879)

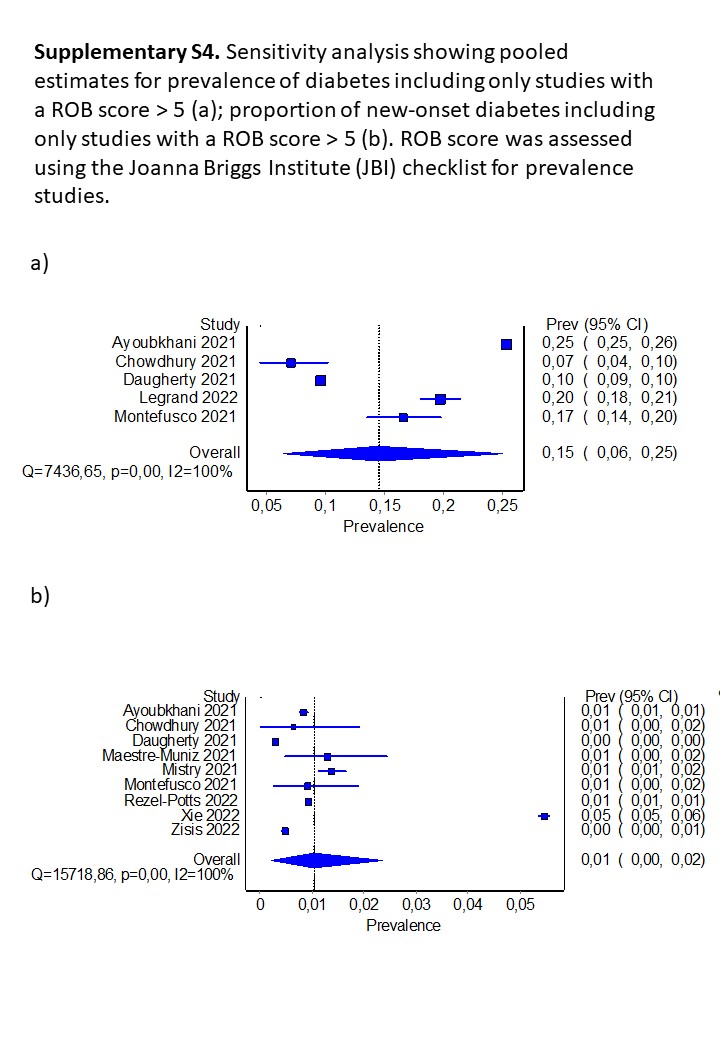

Supplement: Supplementary file 1 [file DataSheet_1.zip › Supplementary S4.JPEG]

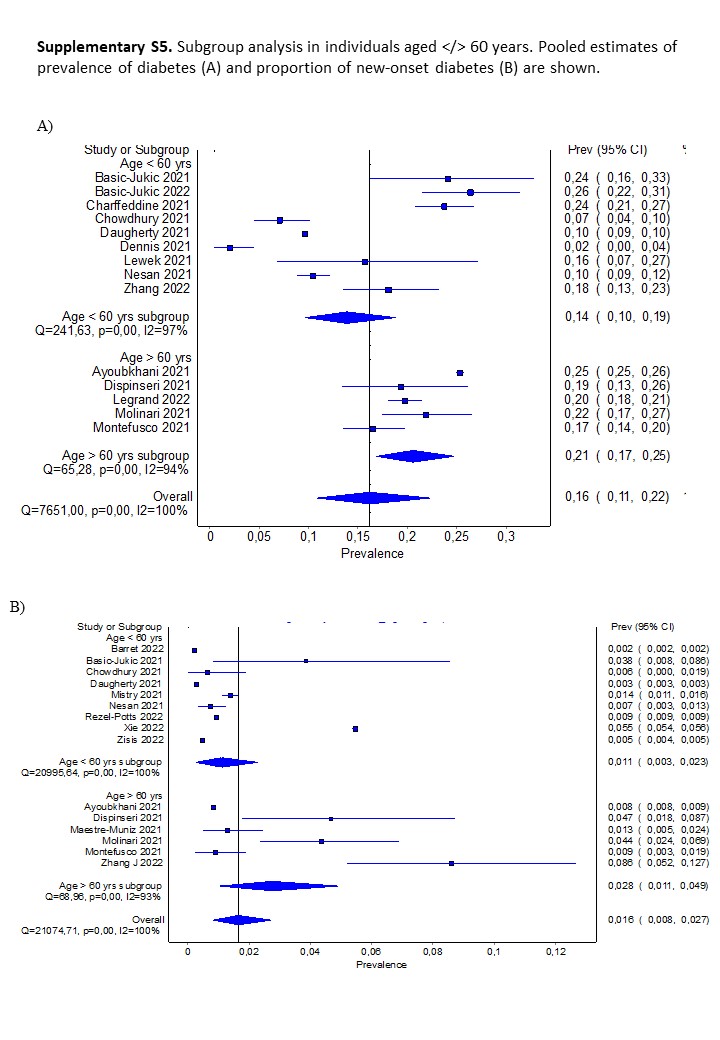

Supplement: Supplementary file 1 [file DataSheet_1.zip › Supplementary S5.JPEG]

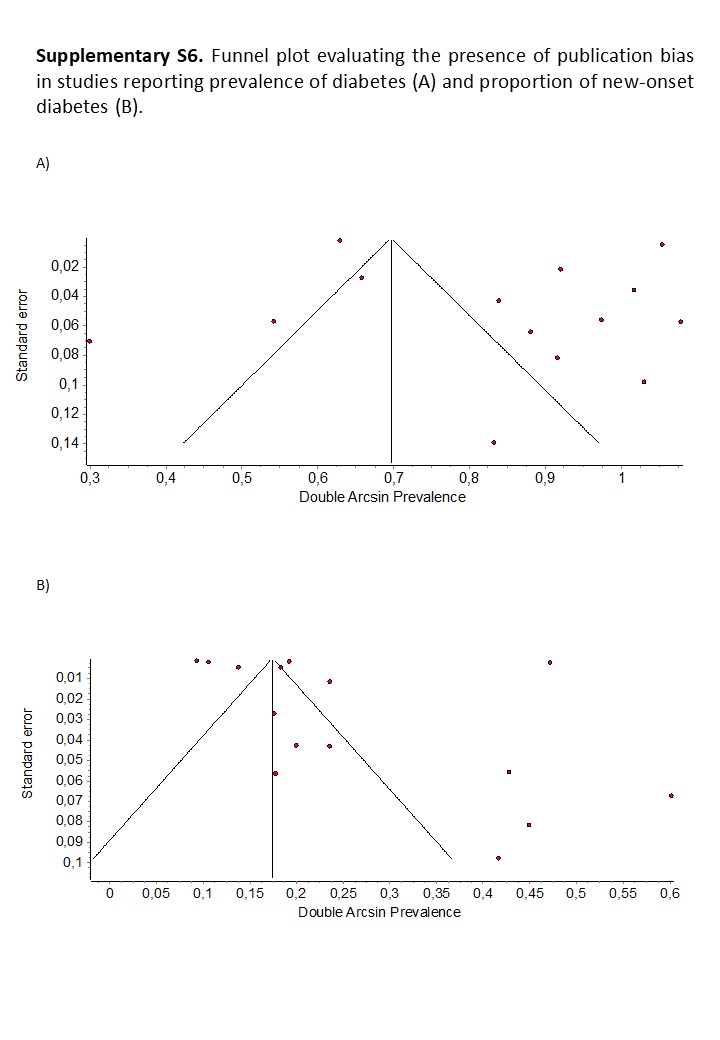

Supplement: Supplementary file 1 [file DataSheet_1.zip › Supplementary S6.JPEG]
